# Methodology of a Cross-sectional Study Evaluating the Impact of a Novel Mobile Care Team on the Prevalence of Ambulatory Care Sensitive Conditions Presenting to Emergency Medical Services

**DOI:** 10.7759/cureus.3369

**Published:** 2018-09-26

**Authors:** Ryan Brown, Alix Carter, Judah Goldstein, Jan L Jensen, Andrew H Travers

**Affiliations:** 1 Emergency Medicine, Dalhousie University, Halifax, CAN

**Keywords:** ambulatory care sensitive conditions, emergency medical services, primary care

## Abstract

Introduction

Hospitalization due to ambulatory care sensitive conditions (ACSC) is often used as a proxy measure for access to primary care. The prevalence of ACSC has not been measured in the prehospital setting. Emergency medical services (EMS) are being used by patients who lack access to primary care for ACSC. Many novel models of care have been implemented within Canada and internationally, utilizing paramedics to ease the burden of poor primary care access. Recently, a mobile care team (MCT) consisting of a paramedic/nurse configuration has been deployed in the community of New Waterford, Nova Scotia. The team responds to low acuity 911 calls and follow-up appointments booked by primary care clinicians. This study will identify the prevalence of patients with ACSC presenting to EMS before and after the implementation of MCT and the differences after the implementation of the MCT.

Methods

Secondary data will be collected from the centralized EMS electronic patient care report (ePCR) database. All patients presenting to the ground ambulance with ACSC during the year prior to MCT implementation, all patients presenting to the ground ambulance with ACSC during the year post-MCT implementation, and all patients presenting to the MCT with ACSC will be included for analysis, allowing for a calculation of ACSC prevalence. Descriptive methods will be used for age, sex, primary care practitioner, and ASCS complaints. Prevalence data will be compared via the chi-squared test. A subgroup analysis of age, sex, and individual presenting conditions will also be analyzed using the chi-squared test. Confounding will be dealt with via multivariate logistic regression.

Results

The study results are pending; however, a literature review reveals a paucity of data on ACSC in EMS.

Conclusions

Due to the paucity of literature surrounding ACSC prevalence in EMS, the methodology developed to study these prevalence rates is a novel protocol of importance to prehospital research and the epidemiology of ACSC more broadly.

## Introduction

Complications that arise from the poor management of chronic conditions are termed ambulatory care sensitive conditions (ACSC) [1-2]. Poor management can be attributed to many factors, including lack of early and ready access to primary care [3]. Complications can be in many forms, including accessing emergency and acute care and admissions to hospital. To that end, improving the management of ACSC should mitigate the unnecessary use of emergency medical services (EMS), emergency department (ED) presentation, and subsequent hospitalizations. A number of conditions are considered ACSC [4-5]. In Canada, this list includes seven conditions: epileptic seizures, chronic obstructive pulmonary disease (COPD), diabetes, hypertension (HTN), angina, congestive heart failure (CHF) (and other pulmonary edema), and asthma, based on the World Health Organization’s International Disease Classification (ICD) codes used by the Canadian Institute for Health Information (CIHI) [3].

While EMS systems are designed to respond to emergencies and paramedics are traditionally trained to assess and treat critically ill patients, patients of lower acuity often utilize EMS for primary care complaints. This is likely because patients or their caregivers view EMS as their only option for accessing urgent care [6]. Due to this and other identified gaps in healthcare, community paramedicine (also termed mobile integrated health programs), a new subspecialty in EMS, has evolved and been implemented in Canada and several other countries [7]. Community paramedicine practitioners are trained, in addition to their traditional emergency training, to treat and manage patients with non-urgent medical issues, minor injuries, and exacerbations of chronic conditions, often in the home without transport to an ED [8]. Alternate models of care utilizing paramedics have been implemented widely in Nova Scotia to ease the burden of poor access to primary health care [9-10]. Collaborative Emergency Centres (CEC), consisting of an onsite nurse/paramedic team with online (via phone) medical oversight, have been implemented in rural areas [11], as have community paramedics working in conjunction with nurse practitioners [9]. Expanded scope paramedics have been implemented to ease the lack of primary care access specific to older adults living in long-term care facilities [12-13]. EMS non-transport may be suggestive of a patient requiring ambulatory care assessment and care, but not emergency care. The current rate of non-transports in the Nova Scotia ground ambulance system is approximately 20% (Abstract: Carrigan S. Prevalence and Characteristics of Non-transported EMS Patients in Nova Scotia. National Association of EMS Physicians Annual Meeting; 2017). It is unknown what the prevalence of ACSC is in EMS calls and community paramedicine models currently utilized in the province.

This study is conducted in the town of New Waterford, Nova Scotia. The Nova Scotia EMS system is operated as a high-performance advanced life support service under the regulation of Emergency Health Services (EHS), a division of the Nova Scotia government Department of Health and Wellness (DHW). The system is evidence-based and research driven and is widely recognized internationally as a leader in the field. Paramedics operate under prospective protocols and clinical practice guidelines with online medical oversight physician consult.

In May of 2013, a cadre of experienced advanced care paramedics and emergency certified registered nurses were trained to respond to low acuity emergency and primary care complaints in New Waterford. This mobile care team (MCT) model was based on a similar team in the United Kingdom as described by Widiatmoko, et al. [14]. The MCT paramedic and nurse work together in a specially outfitted non-transport vehicle to respond to assignments, seeing patients in the community. MCT call dispositions include: treat and discharge, treat and discharge with follow-up, or arrange for EMS transfer to an ED.

The impact of ACSC in this community has not yet been evaluated. It is reasonable to hypothesize improved access to care and treatment of these conditions may lead to a healthier population as a whole in this community as well as overall cost-effectiveness, not only for the local district health authority but for the provincial system in general. It is possible that this model could significantly improve primary health care access and play a part in ED non-conveyance in a system rife with over-crowding.

While community paramedicine literature exists [8-9], this is still very much a developing field of research. ACSC hospitalization is often used as a measure for the effectiveness of and access to primary health care [3,15], however, there is a paucity of literature concerning the paramedic treatment of ACSC and, consequently, the impact on ED visits and subsequent hospital admissions. The aim of the proposed study is to: 1) review literature on ACSC in the prehospital setting and 2) determine the prevalence of ACSC complication (e.g. 911 access, ED access, and/or hospitalization) rate in ground ambulance responses before and after the introduction of the novel MCT program in order to assess the impact of the MCT on ACSC-related calls. The investigators' hypothesis is that there will be a reduction in ACSC complication rate presenting to the ground ambulance.

## Materials and methods

Literature review methods

A comprehensive literature review was conducted in a step-wise manner with the goal of identifying all literature available pertaining to ACSC prevalence in the prehospital setting. A search strategy was devised and executed in relevant databases. A grey literature search was also conducted on Google using the same keywords for searching. The first 100 documents were reviewed for relevance with two documents meeting the criteria. Further to this, references of relevant documents were reviewed and subject matter experts contacted regarding knowledge of seminal articles. All relevant literature was then summarized in the context of this study.

Search Strategy

The literature search was conducted using the MEDLINE, Cumulative Index to Nursing and Allied Health Literature (CINAHL), and Excerpta Medica Database (EMBASE) databases as well as Google Scholar using keyword searching. The keyword search of: “Ambulatory Care Sensitive Conditions” OR “ACSC” OR “Primary Care Sensitive Conditions” OR “PCSC” AND “Emergency Medical Services” OR “EMS” OR “Prehospital” OR “Out of Hospital” OR “Paramedic” OR “Emergency Medical Technician” OR “EMT” OR “Ambulance” AND “Prevalence,” was input into the various databases and Google Scholar with the only limit selected being that of language (English). Terms were combined in various permutations in order to broaden or narrow the search.

Cross-sectional study design and setting

This is a longitudinal, cross-sectional, comparative study making use of secondary data as is appropriate for prevalence estimations. The study population will be drawn from patients in the community of New Waterford, Nova Scotia, Canada, presenting to traditional ground ambulance paramedics as well as the MCT. This model is unique in North America. Ground ambulance and MCT services are provided by Emergency Health Services Nova Scotia.

Sampling, inclusion and exclusion criteria

Patients presenting to the ground ambulance in the New Waterford catchment area with ACSC during the year prior to MCT implementation (October 1, 2012 to September 30, 2013), patients presenting to the MCT from implementation to one year (October 1, 2013, to September 30, 2014) and patients presenting to the ground ambulance from implementation to one year will be included. There will be no limit on age. Patients presenting with any complaint other than ACSC will be excluded. This will allow for the calculation of ACSC prevalence and subsequent evaluation. As 100% of the sample is available via a central electronic health record database, and the number of patients is not expected to be exceedingly large, no sample calculation will be required, as all data will be utilized.

Data collection

Secondary data will be collected from the provincial EMS electronic patient care report (ePCR) database. A data access request will be made to the custodian. Clinical (chief complaint, past medical history, clinical impression, protocol) and demographic (age, gender) data will be queried (Appendix A). Cases will be identified specifically by the paramedic-documented chief complaint, clinical impression, and protocol using the CIHI definition of ACSC [3]. A full chart review of 10% of the charts will be completed by the lead investigator (RB) to ensure the data points are accurately captured.

Data analysis

Descriptive methods will be used for age, sex, current primary care practitioner, and ACSC complaint. The prevalence of ACSC based upon this data will be calculated and compared pre- and post-MCT implementation via the chi-squared test. All data will be categorical, including a subgroup prevalence analysis of age, sex, and individual presenting conditions. This data will also be analyzed using the chi-squared test. As per convention, a p-value of 0.05 or less will be considered statistically significant. Confounding will be dealt with via multivariate logistic regression.

Ethical considerations

Ethics approval will be sought from the Nova Scotia Health Authority Research Ethics Board. There is a risk of a privacy breach, although this risk would be classified as minimal as the research is non-intrusive and there is no risk of identifying cases in the dissemination of results [16]. Appropriate steps will be taken to mitigate this risk as outlined below. A complete census of cases for the pre- and post-implementation periods in question will be conducted and as the sample size is estimated to be greater than 2000 cases, gaining informed consent would not be feasible. This secondary data will be fully de-identified and no dates will be attached to individual cases further to falling within the timeframe for inclusion. All data will be de-identified ensuring anonymity and confidentiality and stored for 7 years on a secured hard-drive in a locked cabinet in a secured office. All patient information will be de-identified prior to chart review for validity purposes.

## Results

The literature review resulted in 151 unique journal articles after de-duplication. A title and abstract review were carried out for all 151 articles with 12 moving on to a full-text review. Of the 12, two articles met the criteria for inclusion, which were a discussion of ACSC in the EMS context. These broad inclusion criteria were decided upon as, based upon expert opinion, there would not be a great deal of literature on the subject.

No articles on overall ACSC prevalence further to the database and grey literature searches were deemed relevant. Due to the paucity of literature specific to ACSC in the context of EMS, the original search terms were re-run in the various databases, substituting permutations of the seven individual ACSCs for “ACSC.” This was found to be too specific a search string so, instead, each individual ACSC was then combined with the “AND” Boolean operator to “prevalence in EMS.” Upon an abstract review, an additional 10 articles were included for full review, bringing the total to 12 studies reviewed from the searches. Furthermore, eight studies pertaining to community paramedicine were also identified and included for review, as the MCT is a form of collaborative community for paramedicine, and data on ACSC in this body of literature may exist.

In total, 20 studies were found to have relevant data pertaining to ACSC complications presenting to EMS; however, none of the studies reviewed report on overall ACSC prevalence in EMS.

As the statistical analysis is pending, there are no results to report in the quantitative portion of the study.

## Discussion

ACSC is a topic of great interest in emergency medicine, as hospitalization rates due to ACSC are often used as a proxy for poor primary health care access. Each ACSC seen in the emergency department costs an average of $280.00 CDN, with each hospitalization costing an average of $5700.00 CDN, an obvious stress on health care resources [3,15]. A recent study focusing on older adults found that approximately 40% of patients transported required no interventions by EMS. While not specific to ACSC, COPD, asthma, CHF, and diabetes were included along with other low acuity presentations [17]. A model that reduces ACSC EMS transports, ED presentations, and subsequent hospitalizations by improving primary care has the potential to positively impact public health in the community. As such, the MCT model will be studied in a rigorous fashion to assess the impact on ACSC.

Some literature exists on individual ACSC conditions (as defined by this study) in both the broad EMS literature and the subspecialty of community paramedicine. Much of this data is confined to subpopulations (frequent users, pediatrics, older adults, etc.) and, therefore, does not reflect the overall prevalence of ACSC in EMS. This data, however, pertaining to specific pathologies in large populations, will be useful in the discussion of the subgroup analysis in the study being conducted.

## Conclusions

Based upon both the review of the peer-reviewed and grey literature, it was concluded that no evidence exists pertaining to overall ACSC prevalence in the EMS setting nor have there been any interventions assessed demonstrating a reduction of ACSC calls in ground ambulance patients. Therefore, it has been concluded that this study is novel in both its methodology and aims and objectives. If this model is found to decrease the call volume of ACSC seen by the ground ambulance, decreases in ED presentation and subsequent hospitalization are likely and must be further investigated. Improved access to care and decreased costs to an already overburdened system will impact favorably on the healthcare system and overall population health.

## Appendices

Appendix A: data points

1. Chief Complaint:

·      Acute Coronary Syndrome

·      Chest Pain (NYD)

·      Diabetic Problem

·      Pulmonary Edema (CHF)

·      Respiratory Arrest

·      Respiratory Distress

·      Seizures

2. Response Type: 

·      Ambulance

·      Mobile Care Team

3. ACSC PMHx: 

·      Epilepsy

·      Seizures

·      Chronic Obstructive Pulmonary Disease

·      Emphysema

·      Chronic Bronchitis

·      Asthma

·      Pulmonary Edema

·      Congestive Heart Failure

·      Hypertension

·      Angina

4. Age (On Day of Call)

5. Sex

6. Primary Care Provider: 

·      Family Physician

·      None

7. Clinical Impression: 

·      Cardiovascular – Angina

·      Cardiovascular – Chest Pain (NYD)

·      Cardiovascular – CHF

·      Cardiovascular – Hypertension

·      Cardiovascular – STEMI; ST-elevation myocardial infarction

·      Hyperglycemic

·      Hypoglycemic

·      Medication Checks

·      Neuro Seizure

·      Respiratory – Arrest

·      Respiratory – Asthma

·      Respiratory - COPD

·      Respiratory – SOB (NYD)

8. Protocol: 

·      Asthma 6289.05

·      COPD 6281.04

·      Ped Resp Distress SOB NYD 6265.01

·      Ped Resp Distress SOB Overview 6264.01

·      Ped Stridor 6266.04

·      Ped Wheeze Bronchospasm 6267.03

·      Pulmonary Edema CHF 6282.05

·      Resp Distress NYD 6283.02

·      Resp Distress SOB Overview 6279.01

·      SOB Stoma/Trach 6284.02

·      Chest Pain (NYD) 6228.01

·      STEMI Restore 6318.01

·      Suspected Cardiac Origin 6229.06

·      Suspected Cardiac Origin Overview 6317.01

·      Ped Seizure 6254.03

·      Seizure 6214.02

·      Hypoglycemia 6212.02

·      Ped Hypoglycemia 6252.01

·      Post Discharge Evaluation of CHF 6506.00

·      Blood Glucose Testing 6509.01

·      Blood Pressure Testing 6508.01

·      MDI Medication Administration 6528.00

·      Medication Reconciliation and Compliance 6507.01

·      Oral Medication Administration 6533.00

·      Patch or Paste Medications 6532.00

9. Master Incident Number

## References

[1] Canadian Institute for Health Information (2012). Waits for emergency department care. Health Care in Canada, 2012: A Focus on Wait Times.

[2] Purdy S, Griffin T, Salisbury C, Sharpe D (2009). Ambulatory care sensitive conditions: terminology and disease coding need to be more specific to aid policy makers and clinicians. Public Health.

[3] (2018). Hospitalizations for ambulatory care sensitive conditions (ACSC): the factors that matter. https://www150.statcan.gc.ca/n1/pub/82-622-x/82-622-x2011007-eng.htm.

[4] Brown AD, Goldacre MJ, Hicks N, Rourke JT, McMurtry RY, Brown JD, Anderson GM (2001). Hospitalization for ambulatory care-sensitive conditions: a method for comparative access and quality studies using routinely collected statistics. Can J Public Health.

[5] Caminal J, Starfield B, Sanchez E, Casanova C, Morales M (2004). The role of primary care in preventing ambulatory care sensitive conditions. Eur J Public Health.

[6] Brown R, Kozicky R (2014). Spotlight on science: improving access through collaboration. Can Paramed.

[7] Wingrove G (2011). International roundtable on community paramedicine. J Emerg Prim Health Care.

[8] Bigham BL, Kennedy SM, Drennan I, Morrison LJ (2013). Expanding paramedic scope of practice in the community: a systematic review of the literature. Prehosp Emerg Care.

[9] Martin-Misener R, Downe-Wamboldt B, Cain E, Girouard M (2009). Cost effectiveness and outcomes of a nurse practitioner-paramedic-family physician model of care: the Long and Brier Islands study. Prim Health Care Res Dev.

[10] (2018). Better care sooner: the plan to improve emergency care in Nova Scotia. https://novascotia.ca/dhw/publications/Better-Care-Sooner-plan.pdf.

[11] Hayden JA, Killian L, Zygmunt A, Babineau J, Martin-Misener R, Jensen JL, Carter AJ (2018). Methods of a multi-faceted rapid knowledge synthesis project to inform the implementation of a new health service model: collaborative emergency centres. Syst Rev.

[12] Jensen JL, Marshall EG, Carter AJ, Boudreau M, Burge F, Travers AH (2016). Impact of a novel collaborative long-term care - EMS model; a before and after cohort analysis of an extended care paramedic program. Prehosp Emerg Care.

[13] Moulton D (2011). Paramedic program reducing emergency room congestion. CMAJ.

[14] Widiatmoko D, Machen I, Dickenson A, Williams J, Kendall S (2008). Developing a new response to non-urgent emergency calls: evaluation of a nurse and paramedic partnership intervention. Prim Health Care Renew Dev.

[15] (2018). Disparities in primary health care experiences among Canadians with ambulatory care sensitive conditions. https://secure.cihi.ca/free_products/PHC_Experiences_AiB2012_E.pdf.

[16] (2014). Privacy and confidentiality. Tri-Council Policy Statement: Ethical Conduct for Research Involving Humans.

[17] Goldstein J, Jensen JL, Carter AJ, Travers AH, Rockwood K (2015). The epidemiology of prehospital emergency responses for older adults in a provincial EMS system. CJEM.

